# Systematic review of endoluminal vacuum‐assisted therapy as salvage treatment for rectal anastomotic leakage

**DOI:** 10.1002/bjs5.50124

**Published:** 2018-12-26

**Authors:** M. Shalaby, S. Emile, H. Elfeki, A. Sakr, S. D. Wexner, P. Sileri

**Affiliations:** ^1^ Department of General Surgery, Colorectal Surgery Unit Mansoura University Mansoura Egypt; ^2^ Department of General Surgery Rome Tor Vergata University Rome Italy; ^3^ Department of Surgery, Colorectal Surgery Unit Aarhus University Aarhus Denmark; ^4^ Department of Colorectal Surgery Cleveland Clinic Florida Weston Florida USA

## Abstract

**Background:**

Endoluminal vacuum‐assisted therapy (EVT) has been introduced recently to treat colorectal anastomotic leakage. The aim of this study was to evaluate the safety and efficacy of EVT in the treatment of anastomotic leakage and rectal stump insufficiency after Hartmann's procedure.

**Methods:**

A systematic search of MEDLINE, Scopus and Cochrane databases was performed using search terms related to EVT and anastomotic leakage or rectal stump insufficiency in line with the PRISMA checklist. Observational studies, RCTs and case series studies published to July 2017 were included. Primary outcomes of the review were the success of EVT, defined as complete or partial healing of the anastomotic defect and associated cavity, and the rate of stoma reversal after EVT. Secondary outcomes included the duration of treatment to complete healing, complications of treatment and the need for further intervention. A meta‐analysis was conducted. The potential effect of clinical confounders on the failure of EVT was investigated using the random‐effects meta‐regression model.

**Results:**

Of 476 articles identified, 17 studies reporting on 276 patients were ultimately included. The weighted mean rate of success was 85·3 (95 per cent c.i. 80·1 to 90·5) per cent, with a median duration from inception of EVT to complete healing of 47 (range 40–105) days. The weighted mean rate of stoma reversal across the studies was 75·9 (64·6 to 87·2) per cent. Twenty‐five patients (9·1 per cent) required additional interventions after EVT. Thirty‐eight patients (13·8 per cent) developed complications. The weighted mean complication rate across the studies was 11·1 (6·0 to 16·2) per cent. Variables significantly associated with failure included preoperative radiotherapy, absence of diverting stoma, complications and male sex.

**Conclusion:**

EVT is associated with a high rate of complete healing of anastomotic leakage and stoma reversal. It is an effective option in appropriately selected patients with anastomotic leakage.

## Introduction

Anastomotic leakage is the most catastrophic surgical complication after rectal cancer surgery, and leads to increased morbidity, additional interventions, hospitalization and death^1^. It adversely affects both oncological and functional outcomes, including a higher than desired rate of permanent stoma^2^ ^3^.

There is no universally accepted management flowchart for anastomotic leakage^4^. Treatment should be individualized based on the patient's general condition, anastomotic defect size and location, indication for primary resection and the presence of a proximal stoma^5^. There has been a paradigm shift in the management of anastomotic leakage from surgical to non‐operative image‐guided and, more recently, endoscopic treatment. Non‐operative management is usually favoured for patients who underwent proximal faecal diversion at the initial operation. In this population, treatment options may include transanal anastomotic tube drainage, percutaneous drainage or recently developed endoscopic procedures, including stent or clip placement or endoluminal vacuum‐assisted therapy (EVT)^6^. In EVT, an open‐pored polyurethane sponge is inserted in the leakage cavity through the anastomotic defect by a flexible endoscope. EVT carries the benefits of being a less invasive approach, ensuring continuous drainage, and promoting granulation and mechanical reduction in the size of the abscess cavity. Endoscopic treatment has been reported to be associated with better outcome, including more frequent preservation of the anastomosis^7^ ^8^.

Although preliminary reports showed promising results for EVT in the treatment of colorectal anastomotic leakage, data determining the superiority of EVT over other forms of conservative treatment in diverted anastomoses are lacking. Clearly defined indications for EVT do not exist^9^ ^10^. The aim of this review was to evaluate the safety and efficacy of EVT in colorectal and coloanal anastomotic leakage and rectal stump insufficiency after Hartmann's procedure, and to investigate the role of EVT in anastomotic salvage.

## Methods

This was a systematic review of studies investigating EVT treatment for anastomotic leakage. The value of vacuum‐assisted closure (VAC) therapy in colorectal surgery has been recognized as it represents an effective, minimally invasive approach to control locally contained anastomotic leakage after pelvic anastomosis^10^. EVT depends on placing an open‐pored sponge into the perirectal cavity through the anastomotic defect by a flexible endoscope. The sponge is connected through a transanally exiting evacuation tube to a vacuum drainage system. Based on the same mechanism as VAC therapy, shrinkage and cleaning of the abscess cavity occur together with closure of the presacral space^9^ ^11^, ^12^, ^13^, ^14^, ^15^, ^16^.

### Search strategy

Registration in the international prospective register of systematic reviews (PROSPERO) preceded the literature search (registration number CRD42016043118). Two investigators independently conducted a computerized search of the literature for studies to evaluate the outcomes of EVT in the treatment of anastomotic leakage after colorectal or coloanal anastomosis and rectal stump insufficiency (opening of rectal stump) following Hartmann's procedure. This review is reported in compliance with the PRISMA checklist^17^.

Electronic databases including PubMed/MEDLINE, Scopus and the Cochrane Central Register of Controlled Trials (CENTRAL) were queried for published and ahead‐of‐publication studies dating from inception of each database to July 2017. The PubMed function ‘related articles’ was used to search for further related articles. In addition, the reference lists of the studies included were screened manually for further potentially relevant articles.

Keywords used in the search process included: ‘anastomosis’, ‘anastomoses’, ‘anastomotic’, ‘leak’, ‘leakage’, ‘insufficiency’, ‘cavity’, ‘colorectal’, ‘coloanal’, ‘Hartmann's', ‘rectal’, ‘stump’, ‘treatment’, ‘endoscopic’, ‘outcome’, ‘endsponge’, ‘vacuum’, ‘endo‐vacuum’, ‘VAC’, ‘endoluminal’, ‘ETVARD’, ‘transanal’, ‘transrectal’, ‘healing’ and ‘closure’. The following Medical Subject Headings (MeSH) terms were also used in the literature search: (endoscopic), (endoluminal), (anastomotic leakage), (treatment outcome) and (healing).

After exclusion of duplicate articles and conference abstracts with no full‐text version the remaining articles were screened and filtered by title and abstract and subsequently by full text. The investigators independently reviewed the full text of the selected studies to verify eligibility.

### Eligibility criteria

All studies evaluating the outcome of EVT in the treatment of anastomotic leakage after colorectal or coloanal anastomosis and rectal stump insufficiency following Hartmann's procedure were considered eligible for inclusion. Comparative and cohort studies were included. Editorials, letters, case reports, reviews and meta‐analyses were excluded. Articles that did not report the primary outcomes of the present review were also excluded. There were no language restrictions.

### Assessment of methodological quality and bias within included studies

Two investigators independently evaluated the methodological quality of each study included in the review. In case of any discrepancy, a third investigator was consulted. The National Institute for Health and Care Excellence (NICE) checklist^18^ was used for assessment of case series and cohort studies; each study was given a score. Quality of the studies was defined as good (score 7–8), fair (score 4–6) or poor (score 3 or less). The quality of comparative studies was assessed using the revised grading system of the Scottish Intercollegiate Guidelines Network (SIGN)^19^, and defined as good (score above 14), fair (score 8–14) or poor (score less than 8).

### Data extraction

Data were extracted and entered into Microsoft Excel® (Microsoft, Redmond, Washington, USA). Data acquisition was performed by two independent teams of investigators. Data extracted from each study included authors' names, year of publication, duration, country and design of the study. Details of study participants were collected, including the number of patients, mean age, male to female ratio, indications for rectal resection, operative intervention, faecal diversion at primary surgery, level of anastomosis, time of detection of anastomotic leakage, and the initial size of the anastomotic cavity.

In addition, information was collected about the equipment used for EVT, type of anaesthesia, indication for EVT as a primary or secondary measure for anastomotic leakage, combined use of EVT with other treatment modalities, number of sponges used in the first treatment session, amount of pressure applied, frequency of sponge changes, total duration of treatment, and total number of sponges used. Information on the incidence of healing, the success of EVT, need for other interventions, treatment complications, stoma reversal and mortality were also collected.

### Outcomes of the review

The primary outcome of the review was the success of EVT, defined as complete or partial healing of the anastomotic defect and associated cavity, and the rate of stoma reversal after EVT. Secondary outcomes included the duration of treatment until complete healing, complications of treatment and the need for further intervention.

### Assessment of publication bias among included studies

**Figure 1:**
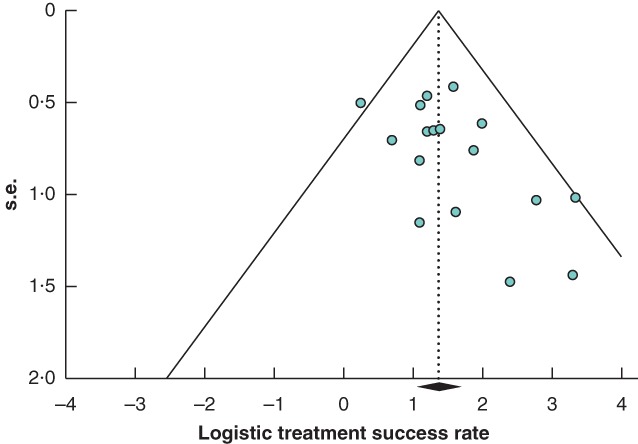
Funnel plot for assessment of publication bias

Publication bias across the studies was assessed using a funnel plot of the standard error of the success rate of EVT for anastomotic leakage *versus* the success rates in the included studies (*Fig*. 1). A straight vertical line in the plot indicates the zone in which 95 per cent of studies should be if there were no publication bias. The Begg and Mazumdar rank correlation test was used to investigate publication bias; Kendall's τb (corrected for ties) was 0·28, with a one‐tailed *P* value of 0·054 and a two‐tailed *P* value of 0·100, indicating no significant publication bias among the studies included.

### Statistical analysis

Data were analysed using SPSS® version 23 (IBM, Armonk, New York, USA). Variables were expressed as mean(s.d.) or median (range) values. Student's *t* test was used to compare quantitative variables. *P <* 0·050 was considered statistically significant.

A meta‐analysis of the rates of treatment success, stoma reversal and complications across the studies was conducted using open‐source, cross‐platform software for advanced meta‐analysis, openMeta[Analyst]™ version 12.11.14 (http://www.cebm.brown.edu/openmeta/). Data were pooled and weighted mean rates with 95 per cent c.i. calculated. Statistical heterogeneity was determined with Cochrane's *Q* test and *I* ^2^ statistics. *I* ^2^ is the proportion of total variation observed between the studies attributable to differences between studies rather than sampling error. Heterogeneity was considered low when *I* ^2^ was less than 25 per cent and high when *I* ^2^ was greater than 75 per cent. If significant statistical heterogeneity was not present a fixed‐effect model was used to pool data, whereas in the case of significant statistical heterogeneity (*P* < 0·100) the binary random‐effects model was employed for pooling of data.

A random‐effects meta‐regression model was used, weighing the studies by their within‐study variance and degree of heterogeneity to determine the predictive factors for failure of EVT in the treatment of anastomotic leakage. Heterogeneity between studies was explored in relation to differences in patient age, sex, creation of a stoma before EVT, radiotherapy, development of complications and duration of treatment. The statistical significance of each examined variable was examined using the slope coefficient (s.e.) and *P* value.

## Results

### Patient and study characteristics

**Figure 2:**
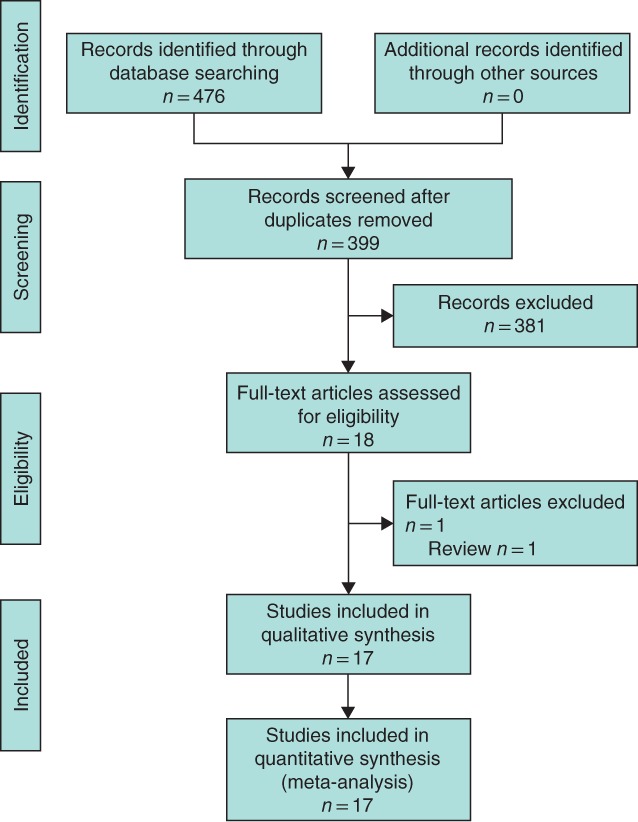
PRISMA flow diagram for the review

After initial screening of 476 articles by title, 77 duplicates were excluded. A further 382 articles were excluded after abstract and full‐text reading, leaving 17 studies^9,11–16,20–29^ published between 2006 and 2017 in the analysis (*Fig*. 2). The studies were performed in Germany (6), the Netherlands (3), Denmark (2), Italy (2), Austria (2), the UK (1) and Turkey (1). No RCTs were retrieved. Two studies had a prospective design and 15 were retrospective. The studies included 276 patients of mean(s.d.) age of 61·6(9·5) (range 37–75) years; the male to female ratio was 2·6 : 1. Characteristics and quality of the studies reviewed are shown in *Table* S1 (supporting information).

### Indications for endoluminal vacuum‐assisted therapy

EVT was used for the management of anastomotic leakage after surgery for colorectal cancer in 209 patients, ulcerative colitis in 21, familial adenomatous polyposis in eight, diverticular disease in six, adenoma in two and endometriosis in one patient. In addition, EVT was used for the management of rectal stump insufficiency in 12 patients, rectal perforation in three, pouch leakage after ileal pouch–anal anastomosis in two, leakage after transanal endoscopic surgery in one patient, leakage after stapled transanal rectal resection in one patient, and treatment of a sacral cavity after abdominoperineal resection in one patient. The indication for EVT in nine patients was not disclosed clearly in one study^29^.

The level of anastomosis was distal colorectal in 135 patients, coloanal in 63 and ileoanal in three. Anastomotic leakage was diagnosed a median of 11·7 (range 6–75) days after the primary operation. Six studies^9^ ^11^, ^24^ ^26^, ^27^ ^29^ diagnosed anastomotic leakage by endoscopy, three^14^ ^21^, ^28^ by contrast studies, and five^12^ ^16^, ^20^ ^22^, ^25^ used both endoscopy and contrast CT. In one study^14^ examination under anaesthesia was used and in another^22^ laparoscopic assessment was employed for confirmation of anastomotic leakage.

### Technical details of endoluminal vacuum‐assisted therapy

Vacuum systems used in the studies included Redyrob® Trans Plus (B. Braun, Melsungen, Germany), Renasys® Go (Smith and Nephew, London, UK), Redon‐Faltenbalg® 250 ml/06–18 Ch (Dahlhausen, Cologne, Germany) and Redovac® 400 ml (B. Braun).

Endosponge therapy was the primary treatment for anastomotic leakage in eight studies^15^ ^16^, ^21^ ^24^, ^25^, ^26^, ^27^, ^28^, and was the primary or secondary treatment in three^9^ ^20^, ^23^. Seven studies^11^ ^12^, ^16^ ^21^, ^24^ ^26^, ^28^ used EVT as a singular treatment for anastomotic leakage, whereas six combined EVT with other treatment methods: intramural fibrin glue in two^9^ ^27^, surgical closure of the defect in two^20^ ^22^ and fibrin glue or stenting in two^15^ ^25^.

Endosponge treatment commenced a median of 15·1 (range 1–173) days after the onset of anastomotic leakage. Treatment was delivered at an endoscopic unit in all studies, except one^25^ in which treatment took place in the operating room. The procedure was done under sedation in nine studies^9^ ^11^, ^20^, ^21^, ^22^ ^25^, ^26^ ^28^, ^29^ and under general anaesthesia in one^14^; two^16^ ^23^ reported the use of sedation or general anaesthesia and four^13^ ^15^, ^24^ ^27^ did not report the type of anaesthesia used.

The median size of the defect treated with EVT was 6 (range 4·7–34·9) cm. Pressure used in the procedure varied from 60 to 200 mmHg, and was up to 700 mmHg in one study^13^. A single sponge was used at the first treatment session, except in one study^9^ that used two sponges to close the defect at the first session. The sponge was changed every 2–3 days in nine studies^9^ ^11^, ^13^, ^14^, ^15^ ^21^, ^23^ ^28^, ^29^ and every 3–4 days in eight^12^ ^16^, ^20^ ^22^, ^24^, ^25^, ^26^, ^27^. The median number of sponges used was 7 (range 3·4–13).

### Outcome of treatment

**Figure 3:**
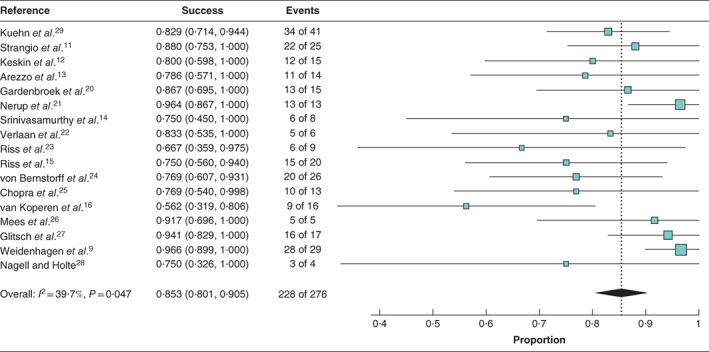
Forest plot for success rate of endoluminal vacuum‐assisted therapy across the studies. A random‐effects model was used for meta‐analysis. Success rates are shown with 95 per cent confidence intervals

In 228 (82·6 per cent) of 276 patients treated with EVT the anastomosis healed completely after treatment (*Table* S2, supporting information). Random‐effects meta‐analysis showed that the weighted mean success rate of EVT was 85·3 (95 per cent c.i. 80·1 to 90·5) per cent (*I* ^2^ = 39·7 per cent) (*Fig*. 3). The duration of treatment to complete healing ranged from 11 to 244 days (median duration 47 (range 40–105) days).

**Figure 4:**
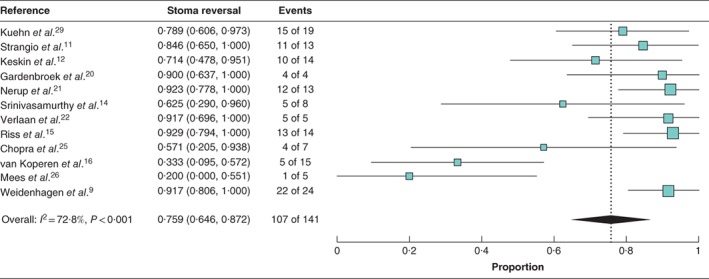
Forest plot for stoma reversal rate after endoluminal vacuum‐assisted therapy across the studies. A random‐effects model was used for meta‐analysis. Stoma reversal rates are shown with 95 per cent confidence intervals

A total of 141 patients had faecal diversion (diversion at initial operation, 132; diversion after diagnosis of anastomotic leakage, 9). Of these 141 patients, 107 (75·9 per cent) who had faecal diversion before or after the start of EVT underwent reversal of stoma following successful treatment. Random‐effects meta‐analysis showed the weighted mean rate of stoma reversal across the studies to be 75·9 (95 per cent c.i. 64·6 to 87·2) per cent (*I* ^2^ = 72·7 per cent) (*Fig*. 4).

Twenty‐five patients (9·1 per cent) required additional interventions after EVT, including Hartmann's procedure (8), faecal diversion (5), CT‐guided drainage of pelvic abscess or collection (4), proctectomy (4), relaparotomy after stoma reversal (2), small bowel resection (1) and insertion of a ureteral stent (1).

**Figure 5:**
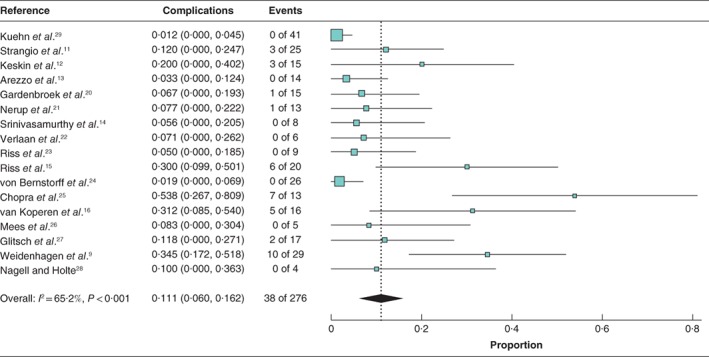
Forest plot for complication rate of endoluminal vacuum‐assisted therapy across the studies. A random‐effects model was used for meta‐analysis. Complication rates are shown with 95 per cent confidence intervals

Thirty‐eight patients (13·8 per cent) developed complications after EVT. Random‐effects meta‐analysis showed that the mean complication rate across the studies was 11·1 (95 per cent c.i. 6·0 to 16·2) per cent) (*I* ^2^ = 65·1 per cent (*Fig*. 5). Complications included: pelvic abscess (16), luminal stenosis (13), bleeding (2), complete dehiscence (2), ileal fistula (1), urethral fistula (1), residual sinus (1), pouch dysfunction (1) and severe pain (1). Ten patients (3·6 per cent) died after EVT from systemic metastasis of colorectal carcinoma.

### Predictors for failure of endoluminal vacuum‐assisted therapy for anastomotic leakage

Variables significantly associated with failure of EVT included preoperative radiotherapy (s.e. 0·163, *P* = 0·018), lack of protective stoma before treatment (s.e. −0·136, *P* = 0·009), development of complications (s.e. 0·194, *P* = 0·002) and male sex (s.e. 0·171, *P* = 0·014) (*Table* S3, supporting information).

Age (s.e. 0·001, *P* = 0·852), date of diagnosis of anastomotic leakage (s.e. 0·001, *P* = 0·393) and duration of treatment (s.e. 0·0001, *P* = 0·760) were not significantly associated with treatment failure.

## Discussion

EVT is a promising, minimally invasive treatment for anastomotic leakage following rectal resection. With a mean success rate of 85 per cent, the need for additional surgery could be reduced significantly. Compared with the current literature, which reports a stoma reversal rate of 30–40 per cent for clinical leakage^30^, the weighted mean rate of stoma reversal across the studies was 75·9 per cent**.** Optimal results may be achieved when endoscopic EVT is offered to patients with distal anastomotic leakage who already have a defunctioning stoma, without sepsis. EVT has a good safety profile with a mean complication rate of approximately 14 per cent. Stenosis is the most common complication, and may be caused by anastomotic leakage rather than by EVT^31^.

The principle of EVT is continuous or intermittent suction and drainage via an open‐cell polyurethane sponge placed on or into a wound with the application of controlled negative pressure^9^ ^26^, ^28^ ^29^, ^32^. The precise mechanism of accelerated healing power is still unclear. It may, however, be attributed to fluid removal, oedema reduction and increased local blood perfusion, which in turn reduce bacterial colonization and stimulate granulation tissue growth^9^ ^23^, ^26^ ^28^, ^32^ ^33^.

Factors associated with failure of EVT included neoadjuvant therapy, lack of a protective stoma before treatment, complications related to EVT and male sex. Most of these are well known risk factors for anastomotic leakage in general^34^. The association between failure of EVT and lack of a protective stoma might be explained by contamination of the sponge with the bacterial content of the stool^26^ or sponge obstruction by the impacted stool^25^. In addition, in the absence of a stoma at the time of therapy the sponge has to drain luminal content as well as air, so it is more likely to lose the adequate negative suction in the cavity^20^, which in turn may increase the chance of sponge dislocation^26^.

There has been a debate on the long‐term results, particularly concerning the stability of EVT‐derived granulation tissue and the possibility of developing a recurrent abscess. Riss and colleagues^15^ assessed the long‐term results after primary successful EVT in a multicentre study and found that 25 per cent of patients developed recurrent abscesses after median follow‐up of 17 months. Oncological and functional outcomes must be investigated further. Most studies included in the current review did not report long‐term outcomes.

The use of EVT in this review was not standardized as it was applied for a myriad of indications and at different anastomotic heights. While some investigators found that higher colorectal anastomoses could be treated with EVT with no difficulty^26^, others^20^ ^22^, ^28^ found it difficult to apply, recommending to avoid EVT in higher anastomoses, and postulated that EVT at higher levels might increase the incidence of small bowel fistula. Cavity size did not seem to predict outcome, as EVT proved suitable for small as well as large leakage cavities. The sponge can be compressed to approximately 8 mm in diameter for smaller cavities^26^, and more than one sponge can be inserted at the initial therapy session^9^.

The timing of EVT can significantly influence success. Weidenhagen and co‐workers^9^ reported a high success rate when EVT was started within 6 weeks of the initial operation. These results were replicated by van Koperen *et al*.^16^, with a success rate of 75 per cent (6 of 8 patients) for EVT of anastomotic leakage commenced within 6 weeks of the initial surgery, compared with 38 per cent (3 of 8) for later EVT.

EVT treatment is more versatile than alternative endoscopic treatments such as stent insertion and fibrin glue. Although EVT can be applied regardless of the level of anastomosis and size of abscess cavity, stent insertion should be avoided in low anastomotic leakage as this has a higher incidence of patient discomfort and possibility of migration, and can be used only in abscess cavities smaller than 2 cm^35^. As stents do not permit internal drainage, perirectal abscess following stenting should also be treated by surgical or percutaneous drainage. Uncovered metal stents may be embedded in the rectal wall, and may perforate^36^. Fibrin glue is limited to leaks of less than 3 mm in diameter without any connecting cavities or abscess^25^.

This review has a number of limitations related to the available literature. These include small sample size. The design of most studies was retrospective. Despite the moderate statistical heterogeneity among studies, clinical heterogeneity was significant, including methods, indications and timing. It is therefore not possible to compare these studies on all endpoints. Long‐term oncological and functional outcomes are awaited.

## Disclosure

The authors declare no conflict of interest.

## Supporting information


**Table S1** Characteristics of included studies
**Table S2** Technical details and outcome of EVT treatment in the included studies
**Table S3** Predictors for success of EVT therapy of anastomotic leakage
