# Supplementary material for: Systematic review of endoluminal vacuum‐assisted therapy as salvage treatment for rectal anastomotic leakage
Source: BJS Open. 2018 Dec 26;3(2):153–60. doi: 10.1002/bjs5.50124 (PMC6433422; doi:10.1002/bjs5.50124)
Supplement: Supplementary file 1 — Table S1 Characteristics of included studies Table S2 Technical details and outcome of EVT treatment in the included studies Table S3 Predictors for success of EVT therapy of anastomotic leakage [file BJS5-3-153-s001.docx]

**BJS5_50124**

**Systematic review of endoluminal vacuum-assisted therapy as salvage treatment for rectal anastomotic leakage**

**M. Shalaby, S. Emile, H. Elfeki, A. Sakr, S. D. Wexner and P. Sileri**

**Table 1 Characteristics of the studies included**

| **Study** | **Type** | **Number** | **Males (%)** | **Median age in years (range)** | **Anastomotic level** | **Study quality** |
| --- | --- | --- | --- | --- | --- | --- |
| **Kuehn et al, ^29^ 2016** | Retrospective | 41 | 31 (75.6) | 70 (29-91) | NA | 5 (fair) |
| **Strangio et al, ^11^ 2015** | Prospective | 25 | 18 (72) | 67 (37-89) | NA | 7 (good) |
| **Keskin et al, ^12^ 2015** | Retrospective | 15 | 7 (46.6) | 55 (25-72) | Low pelvic colorectal | 5 (fair) |
| **Arezzo et al, ^13^ 2015** | Retrospective | 14 | 7 (50) | 68 (55-85) | Low pelvic colorectal | 6 (fair) |
| **Gardenbroek et al, ^20^ 2015** | Prospective | 15 | 12 (80) | 37 (25-56) | Low pelvic colorectal | 9 (fair) |
| **Nerup et al, ^21^ 2013** | Retrospective | 13 | 11 (84.6) | 64 (36-71) | Coloanal | 6 (fair) |
| **Srinivasamurthy et al, ^14^ 2013** | Retrospective | 8 | 7 (87.5) | 66.5 (45-79) | Low pelvic colorectal (n=6)  Coloanal(n=1)  Ileoanal(n=1) | 5 (fair) |
| **Verlaan et al, ^22^ 2011** | Prospective | 6 | 5 (83.3) | 50.2 (29-68) | Low pelvic colorectal | 6 (fair) |
| **Riss et al, ^23^ 2010** | Retrospective | 9 | 5 (55.5) | 63.5 (50-71) | NA | 5 (fair) |
| **Riss et al, ^15^ 2010** | Prospective | 20 | 13 (65) | 66.3 (54.8-91.2) | Coloanal | 8 (good) |
| **von Bernstorff et al, ^24^ 2009** | Prospective | 26 | 21 (80.7) | 62.4 (42-84) | Low pelvic colorectal | 6 (fair) |
| **Chopra et al, ^25^ 2009** | Retrospective | 13 | NA | 65 (33-83) | Coloanal | 5 (fair) |
| **van Koperen et al, ^16^ 2009** | Retrospective | 16 | 9 (56.2) | 64 (19-78) | Coloanal/ileoanal | 6 (fair) |
| **Mees et al, ^26^ 2008** | Retrospective | 5 | 4 (80) | 46 (33-65) | Low pelvic colorectal/ IPAA | 6 (fair) |
| **Glitsch et al, ^27^ 2008** | Prospective | 17 | 14 (82.3) | 61.2 (42-84) | Low pelvic colorectal | 6 (fair) |
| **Weidenhagen et al, ^9^ 2008** | Retrospective | 29 | 24 (82.7) | 66.7 (42-79) | Low pelvic colorectal | 5 (fair) |
| **Nagell & Holte, ^28^ 2006** | Retrospective | 4 | NA | 75 (73-78) | Low pelvic colorectal | 9 (fair) |
| **Total** | ------ | 276 | 188 | 61.6 | ------ | ------ |

**Table 2 Technical details and outcome of EVT treatment in the studies included**

| **Study** | **Using adjunct treatment** | **Frequency of changing sponge** | **Median duration of therapy in days (range)** | **Success of treatment (%)** | **Complications**  **(%)** | **Mortality**  **(%)** |
| --- | --- | --- | --- | --- | --- | --- |
| **Kuehn et al, ^29^ 2016** | None | Every 3 days | 244 (152-488) | 34/41 (82.9) | 0 | 0 |
| **Strangio et al, ^11^ 2015** | None | Every 2-3 days | 34 (1-221) | 22/25 (88) | 3/25 (12) | 0 |
| **Keskin et al, ^12^ 2015** | None | Every 3-4 days | NA | 12/15 (80) | 3/15 (20) | 3 (20) |
| **Arezzo et al, ^13^ 2015** | None | 2-3 times per week | 40.5 (8-114) | 11/14 (78.5) | 0 | 0 |
| **Gardenbroek et al, ^20^ 2015** | Surgical closure of defect | Every 3-4 days | 48 (25-103) | 13/15 (86.6) | 1/15 (6.6) | 0 |
| **Nerup et al, ^21^ 2013** | None | Every 2-3 days | 18 (3-40) | 13/13 (100) | 1/13 (7.6) | 0 |
| **Srinivasamurthy et al, ^14^ 2013** | None | NA | 26 (7-49) | 6/8 (75) | 0 | 0 |
| **Verlaan et al, ^22^ 2011** | Suturing (1) Endoclip (1) | Every 3-4 days | 13.8 (5-28) | 5/6 (83.3) | 0 | 0 |
| **Riss et al, ^23^ 2010** | None | Every 2-3 | 21 | 6/9 (66.6) | 0 | 1 (11.1) |
| **Riss et al, ^15^ 2010** | Stent - Fibrin glue | Every 2-3 days | 21 | 15/20 (75) | 6/20 (30) | 5 (25) |
| **von Bernstorff et al, ^24^ 2009** | None | Every 2-4 days | 21.5 (4-88) | 20/26 (76.9) | 0 | 0 |
| **Chopra et al, ^25^ 2009** | Stent (6) Fibrin glue (2) | Every 3-5 days | 11 (7-14) | 10/13 (76.9) | 7/13 (53.8) | 0 |
| **van Koperen et al, ^16^ 2009** | None | Every 3-4 days | 40 (28-90) | 9/16 (56.2) | 5/16 (31.2) | 0 |
| **Mees et al, ^26^ 2008** | None | Every 3 days | 27 (18-37) | 5/5 (100) | 0 | 0 |
| **Glitsch et al, ^27^ 2008** | Intramural fibrin glue injection | Every 2days then every 3-4 days | 21.4 (4-88) | 16/17 (94.1) | 2/17 (11.7) | 0 |
| **Weidenhagen et al, ^9^ 2008** | Intramural fibrin glue injection | Every 2-3 days | 34.4 (4-79) | 28/29 (96.5) | 10/29 (34.4) | 0 |
| **Nagell & Holte, ^28^ 2006** | None | Every 2-3 days | 51 (43-195) | 3/4 (75) | 0 | 1 (25) |
| **Total** | ------ | ------ | ------ | 228/276 (82.6) | 38/276 (13.8) | 10/276 (3.6) |

**Table 3 Predictors for success of EVT therapy of AL**

| **Study** | **Median age in years (range)** | **Male patients (%)** | **Creation of stoma before treatment (%)**  **(Both at the original operation or after diagnosing AL)** | **Preoperative radiation therapy (%)** | **Median duration of treatment in days (range)** | **Failure of closure of anastomotic defect (%)** |
| --- | --- | --- | --- | --- | --- | --- |
| **Kuehn et al, ^29^ 2016** | 70 (29-91) | 31/41 (75.6) | 19/41 (46.3) | 31/41 (75.6) | 244 (152-488) | 7/41 (16.1) |
| **Strangio et al, ^11^ 2015** | 67 (37-89) | 18/25 (72) | 13/25 (52) | 8/25 (32) | 34 (1-221) | 3/25 (12) |
| **Keskin et al, ^12^ 2015** | 55 (25-72) | 7/15 (46.6) | 14/15 (93.3) | 6/15 (40) | NA | 3/15 (20) |
| **Arezzo et al, ^13^ 2015** | 68 (55-85) | 7/14 (50) | 8/14 (57.1) | 7/14 (50) | 40.5 (8-114) | 3/14 (21.5) |
| **Gardenbroek et al, ^20^ 2015** | 37 (25-56) | 12/15 (80) | 4/15 (26.6) | 0 | 48 (25-103) | 2/15 (13.4) |
| **Nerup et al, ^21^ 2013** | 64 (36-71) | 11/13 (84.6) | 13/13 (100) | 6/13 (46.1) | 18 (3-40) | 0 |
| **Srinivasamurthy et al, ^14^ 2013** | 66.5 (45-79) | 7/8 (87.5) | 8 /8(100) | 7 /8(87.5) | 26 (7-49) | 2/8 (25) |
| **Verlaan et al, ^22^ 2011** | 50.2 (29-68) | 5/6 (83.3) | 5 /6 (83.3) | 1/6 (16.6) | 13.8 (5-28) | 1/6 (16.7) |
| **Riss et al, ^23^ 2010** | 63.5 (50-71) | 5/9 (55.5) | 4/9 (44.4) | 4/9 (44.4) | 21 | 3/9 (33.4) |
| **Riss et al, ^15^ 2010** | 66.3 (54.8-91.2) | 13/20 (65) | 14/20 (70) | 6/20 (30) | 21 | 5/20 (25) |
| **von Bernstorff et al, ^24^ 2009** | 62.4 (42-84) | 21/26 (80.7) | 20/26 (76.9) | 14/26 (53.8) | 21.5 (4-88) | 6/26 (23.1) |
| **Chopra et al, ^25^ 2009** | 65 (33-83) | NA | 7/17 (53.8) | 6/17 (46.1) | 11 (7-14) | 3/17 (23.1) |
| **van Koperen et al, ^16^ 2009** | 64 (19-78) | 9/16 (56.2) | 15/16 (93.7) | 11/16 (68.7) | 40 (28-90) | 7/16 (43.8) |
| **Mees et al, ^26^ 2008** | 46 (33-65) | 4/5 (80) | 5/5 (100) | 0 | 27 (18-37) | 0 |
| **Glitsch et al, ^27^ 2008** | 61.2 (42-84) | 14/17 (82.3) | 13/17 (76.5) | 9/17 (52.9) | 21.4 (4-88) | 1/17 (5.9) |
| **Weidenhagen et al, ^9^ 2008** | 66.7 (42-79) | 24/29 (82.7) | 24/29 (82.7) | 9/29 (31) | 34.4 (4-79) | 1/29 (3.5) |
| **Nagell & Holte, ^28^ 2006** | 75 (73-78) | NA | 4/4 (100) | 1/4 (25) | 51 (43-195) | 1/4 (25) |
| **Total** | 61.6 | 188/276 | 190/276 | 126/276 | ------ | 48/276 |
